# Supplementary material for: Modelling Skylarks (Alauda arvensis) to Predict Impacts of Changes in Land Management and Policy: Development and Testing of an Agent-Based Model
Source: PLoS One. 2013 Jun 6;8(6):e65803. doi: 10.1371/journal.pone.0065803 (PMC3675089; doi:10.1371/journal.pone.0065803)
Supplement: Supporting Information S4 — The skylark ODdox as a zipped archive. (ZIP) [file pone.0065803.s004.zip › Skylark_ODdox/class_user_defined_farm3.html]

ALMaSS Skylark ODdox: UserDefinedFarm3 Class Reference


ALMaSS Skylark ODdox
 2.0


- Main Page
- Related Pages
- Classes
- Files

- Class List
- Class Index
- Class Hierarchy
- Class Members

Public Member Functions

UserDefinedFarm3 Class Reference

A farm that can have its rotation defined by the user at runtime.
More...

`#include <farm.h>`

List of all members.

|  |  |
| --- | --- |
| Public Member Functions | |
|  | UserDefinedFarm3 (void) |
| Public Member Functions inherited from Farm | |
| void | AddField (LE \*a\_newfield) |
|  | Adds a field to a farm. |
| void | AddNewEvent (TTypesOfVegetation a\_event, long a\_date, LE \*a\_field, int a\_todo, long a\_num, bool a\_lock, int a\_start, bool a\_first\_year, TTypesOfVegetation a\_crop) |
|  | Adds an event to the event queue for a farm. |
| virtual bool | AutumnHarrow (LE \*a\_field, double a\_user, int a\_days) |
|  | Carry out a harrow event in the autumn on a\_field. |
| virtual bool | AutumnPlough (LE \*a\_field, double a\_user, int a\_days) |
|  | Carry out a ploughing event in the autumn on a\_field. |
| virtual bool | AutumnRoll (LE \*a\_field, double a\_user, int a\_days) |
|  | Carry out a roll event in the autumn on a\_field. |
| virtual bool | AutumnSow (LE \*a\_field, double a\_user, int a\_days) |
|  | Carry out a sowing event in the autumn on a\_field. |
| virtual bool | BurnStrawStubble (LE \*a\_field, double a\_user, int a\_days) |
|  | Burn stubble on a\_field. |
| virtual bool | CattleIsOut (LE \*a\_field, double a\_user, int a\_days, int a\_max) |
|  | Generate a 'cattle\_out' event for every day the cattle are on a\_field. |
| virtual bool | CattleIsOutLow (LE \*a\_field, double a\_user, int a\_days, int a\_max) |
|  | Generate a 'cattle\_out\_low' event for every day the cattle are on a\_field. |
| virtual bool | CattleOut (LE \*a\_field, double a\_user, int a\_days) |
|  | Start a grazing event on a\_field today. |
| virtual bool | CattleOutLowGrazing (LE \*a\_field, double a\_user, int a\_days) |
|  | Start a extensive grazing event on a\_field today. |
| virtual bool | CutToHay (LE \*a\_field, double a\_user, int a\_days) |
|  | Carry out hay cutting on a\_field. |
| virtual bool | CutToSilage (LE \*a\_field, double a\_user, int a\_days) |
|  | Cut vegetation for silage on a\_field. |
| virtual bool | CutWeeds (LE \*a\_field, double a\_user, int a\_days) |
|  | Carry out weed topping on a\_field. |
| virtual bool | DeepPlough (LE \*a\_field, double a\_user, int a\_days) |
|  | Carry out a deep ploughing event on a\_field. |
| bool | DoIt (int a\_probability) |
|  | Return chance out of 100. |
| virtual bool | FA\_AmmoniumSulphate (LE \*a\_field, double a\_user, int a\_days) |
|  | Apply ammonium supahte to a\_field owned by an stock farmer. |
| virtual bool | FA\_GreenManure (LE \*a\_field, double a\_user, int a\_days) |
|  | Spread green manure on a\_field owned by an stock farmer. |
| virtual bool | FA\_Manure (LE \*a\_field, double a\_user, int a\_days) |
|  | Spread manure on a\_field owned by an stock farmer. |
| virtual bool | FA\_NPK (LE \*a\_field, double a\_user, int a\_days) |
|  | Apply NPK fertilizer to a\_field owned by an stock farmer. |
| virtual bool | FA\_PK (LE \*a\_field, double a\_user, int a\_days) |
|  | Apply PK fertilizer to a\_field owned by an stock farmer. |
| virtual bool | FA\_Sludge (LE \*a\_field, double a\_user, int a\_days) |
|  | Spread sewege sludge on a\_field owned by an stock farmer. |
| virtual bool | FA\_Slurry (LE \*a\_field, double a\_user, int a\_days) |
|  | Spready slurry on a\_field owned by an stock farmer. |
|  | Farm (void) |
|  | Farm constructor - creates an instance of each possible crop type. |
| virtual bool | FP\_GreenManure (LE \*a\_field, double a\_user, int a\_days) |
|  | Spread green manure on a\_field owned by an arable farmer. |
| virtual bool | FP\_LiquidNH3 (LE \*a\_field, double a\_user, int a\_days) |
|  | Apply liquid ammonia fertilizer to a\_field owned by an arable farmer. |
| virtual bool | FP\_ManganeseSulphate (LE \*a\_field, double a\_user, int a\_days) |
|  | Apply Manganse Sulphate to a\_field owned by an arable farmer. |
| virtual bool | FP\_Manure (LE \*a\_field, double a\_user, int a\_days) |
|  | Spread manure on a\_field owned by an arable farmer. |
| virtual bool | FP\_NPK (LE \*a\_field, double a\_user, int a\_days) |
|  | Apply NPK fertilizer, on a\_field owned by an arable farmer. |
| virtual bool | FP\_NPKS (LE \*a\_field, double a\_user, int a\_days) |
|  | Apply NPKS fertilizer, on a\_field owned by an arable farmer. |
| virtual bool | FP\_PK (LE \*a\_field, double a\_user, int a\_days) |
|  | Apply PK fertilizer, on a\_field owned by an arable farmer. |
| virtual bool | FP\_Sludge (LE \*a\_field, double a\_user, int a\_days) |
|  | Spread sewege on a\_field owned by an arable farmer. |
| virtual bool | FP\_Slurry (LE \*a\_field, double a\_user, int a\_days) |
|  | Apply slurry to a\_field owned by an arable farmer. |
| virtual bool | FungicideTreat (LE \*a\_field, double a\_user, int a\_days) |
|  | Apply fungicide to a\_field. |
| int | GetArea (void) |
|  | Returns the area of arable fields owned by that farm. |
| int | GetFarmNumber (void) |
| int | GetIntensity (void) |
| TTypesOfFarm | GetType (void) |
| virtual bool | Glyphosate (LE \*a\_field, double a\_user, int a\_days) |
| virtual bool | GrowthRegulator (LE \*a\_field, double a\_user, int a\_days) |
|  | Apply growth regulator to a\_field. |
| virtual bool | Harvest (LE \*a\_field, double a\_user, int a\_days) |
|  | Carry out a harvest on a\_field. |
| virtual bool | HayBailing (LE \*a\_field, double a\_user, int a\_days) |
|  | Carry out hay bailing on a\_field. |
| virtual bool | HayTurning (LE \*a\_field, double a\_user, int a\_days) |
|  | Carry out hay turning on a\_field. |
| virtual bool | HerbicideTreat (LE \*a\_field, double a\_user, int a\_days) |
|  | Apply herbicide to a\_field. |
| virtual bool | HillingUp (LE \*a\_field, double a\_user, int a\_days) |
|  | Do hilling up on a\_field, probably of potatoes. |
| virtual void | InitiateManagement (void) |
|  | Kicks off the farm's management. |
| virtual bool | InsecticideTreat (LE \*a\_field, double a\_user, int a\_days) |
|  | Apply insecticide to a\_field. |
| bool | IsStockFarmer (void) |
| virtual void | MakeStockFarmer (void) |
| virtual void | Management (void) |
|  | Starts the main management loop for the farm and performs some error checking. |
| virtual bool | Molluscicide (LE \*a\_field, double a\_user, int a\_days) |
|  | Apply molluscidie to a\_field. |
| virtual bool | PigsAreOut (LE \*a\_field, double a\_user, int a\_days) |
|  | Start a pig grazing event on a\_field today or soon. |
| virtual bool | PigsAreOutForced (LE \*a\_field, double a\_user, int a\_days) |
|  | Start a pig grazing event on a\_field today - no exceptions. |
| virtual bool | PigsOut (LE \*a\_field, double a\_user, int a\_days) |
|  | Generate a 'pigs\_out' event for every day the cattle are on a\_field. |
| virtual bool | ProductApplication0 (LE \*a\_field, double a\_user, int a\_days) |
|  | Apply test pesticide to a\_field. |
| virtual bool | ProductApplication1 (LE \*a\_field, double a\_user, int a\_days) |
|  | Apply test pesticide to a\_field. |
| void | RemoveField (LE \*a\_field) |
|  | Removes a field to a farm. |
| virtual bool | RowCultivation (LE \*a\_field, double a\_user, int a\_days) |
|  | Carry out a harrowing between crop rows on a\_field. |
| void | SetFarmNumber (int a\_farm\_num) |
| virtual bool | SleepAllDay (LE \*a\_field, double a\_user, int a\_days) |
|  | Nothing to to today on a\_field. |
| virtual bool | SpringHarrow (LE \*a\_field, double a\_user, int a\_days) |
|  | Carry out a harrow event in the spring on a\_field. |
| virtual bool | SpringPlough (LE \*a\_field, double a\_user, int a\_days) |
|  | Carry out a ploughing event in the spring on a\_field. |
| virtual bool | SpringRoll (LE \*a\_field, double a\_user, int a\_days) |
|  | Carry out a roll event in the spring on a\_field. |
| virtual bool | SpringSow (LE \*a\_field, double a\_user, int a\_days) |
|  | Carry out a sowing event in the spring on a\_field. |
| virtual bool | StrawChopping (LE \*a\_field, double a\_user, int a\_days) |
|  | Carry out straw chopping on a\_field. |
| virtual bool | Strigling (LE \*a\_field, double a\_user, int a\_days) |
|  | Carry out a mechanical weeding on a\_field. |
| virtual bool | StriglingSow (LE \*a\_field, double a\_user, int a\_days) |
|  | Carry out a mechanical weeding followed by sowing on a\_field. |
| virtual bool | StubbleHarrowing (LE \*a\_field, double a\_user, int a\_days) |
|  | Carry out stubble harrowing on a\_field. |
| virtual bool | Swathing (LE \*a\_field, double a\_user, int a\_days) |
|  | Cut the crop on a\_field and leave it lying (probably rape) |
| virtual bool | SynInsecticideTreat (LE \*a\_field, double a\_user, int a\_days) |
|  | Apply special insecticide to a\_field. |
| TTypesOfVegetation | TranslateCropCodes (std::string &str) |
| virtual bool | Trial\_Control (LE \*a\_field, double a\_user, int a\_days) |
|  | Special pesticide trial functionality. |
| virtual bool | Trial\_PesticideTreat (LE \*a\_field, double a\_user, int a\_days) |
|  | Special pesticide trial functionality. |
| virtual bool | Trial\_PesticideTreat\_GS (LE \*a\_field, double a\_user, int a\_days) |
|  | Special pesticide trial functionality. |
| virtual bool | Trial\_ToxicControl (LE \*a\_field, double a\_user, int a\_days) |
|  | Special pesticide trial functionality. |
| virtual bool | Water (LE \*a\_field, double a\_user, int a\_days) |
|  | Carry out a watering on a\_field. |
| virtual bool | WinterPlough (LE \*a\_field, double a\_user, int a\_days) |
|  | Carry out a ploughing event in the winter on a\_field. |
| virtual | ~Farm (void) |
|  | Farm destructor - deletes all crop instances and empties event queues. |

|  |  |
| --- | --- |
| Additional Inherited Members | |
| Protected Member Functions inherited from Farm | |
| void | CheckRotationManagementLoop (FarmEvent \*ev) |
| virtual int | GetFirstCropIndex (TTypesOfLandscapeElement a\_type) |
|  | Gets the first crop for the farm. |
| int | GetFirstDate (TTypesOfVegetation a\_tov) |
|  | Gets the start date for a crop type. |
| virtual int | GetNextCropIndex (int a\_rot\_index) |
|  | Returns the next crop in the rotation. |
| int | GetNextCropStartDate (LE \*a\_field, TTypesOfVegetation &a\_curr\_veg) |
|  | Returns the start date of the next crop in the rotation. |
| void | HandleEvents (void) |
|  | If there are events to carry out do this, and perhaps start a new crop. |
| bool | LeSwitch (FarmEvent \*ev) |
|  | Call do function for any crop with an outstanding event. Signal if the crop has terminated. |
| void | ReadRotation (std::string fname) |
|  | Reads a rotation file into the rotation. |
| Protected Attributes inherited from Farm | |
| AgroChemIndustryCereal \* | m\_agrochemindustrycereal |
| Carrots \* | m\_carrots |
| CloverGrassGrazed1 \* | m\_CGG1 |
| CloverGrassGrazed2 \* | m\_CGG2 |
| int | m\_farm\_num |
| TTypesOfFarm | m\_farmtype |
| FieldPeas \* | m\_fieldpeas |
| FieldPeasStrigling \* | m\_fieldpeasstrigling |
| vector< LE \* > | m\_fields |
| Fodderbeet \* | m\_fodderbeet |
| FodderGrass \* | m\_foddergrass |
| int | m\_intensity |
| Maize \* | m\_maize |
| MaizeSilage \* | m\_maizesilage |
| MaizeStrigling \* | m\_maizestrigling |
| Oats \* | m\_oats |
| OBarleyPeaCloverGrass \* | m\_OBarleyPCG |
| OCarrots \* | m\_ocarrots |
| OCloverGrassGrazed1 \* | m\_OCGG1 |
| OCloverGrassGrazed2 \* | m\_OCGG2 |
| OCloverGrassSilage1 \* | m\_OCGS1 |
| OFieldPeas \* | m\_ofieldpeas |
| OFieldPeasSilage \* | m\_ofieldpeassilage |
| OFirstYearDanger \* | m\_ofirstyeardanger |
| OGrazingPigs \* | m\_ograzingpigs |
| OMaizeSilage \* | m\_omaizesilage |
| OOats \* | m\_ooats |
| OPermanentGrassGrazed \* | m\_opermgrassgrazed |
| OPotatoes \* | m\_opotatoes |
| OSBarleySilage \* | m\_osbarleysilage |
| OSpringBarley \* | m\_ospringbarley |
| OSpringBarleyExt \* | m\_ospringbarleyext |
| OSpringBarleyPigs \* | m\_ospringbarleypigs |
| OWinterBarley \* | m\_owinterbarley |
| OWinterBarleyExt \* | m\_owinterbarleyext |
| OWinterRape \* | m\_owinterrape |
| OWinterRye \* | m\_owinterrye |
| OWinterWheatUndersown \* | m\_owinterwheatundersown |
| OWinterWheatUndersownExt \* | m\_owinterwheatundersownext |
| PermanentSetAside \* | m\_permanentsetaside |
| vector< PermCropData > | m\_PermCrops |
| PermanentGrassGrazed \* | m\_permgrassgrazed |
| PermanentGrassLowYield \* | m\_permgrasslowyield |
| PermanentGrassTussocky \* | m\_permgrasstussocky |
| Potatoes \* | m\_potatoes |
| PotatoesIndustry \* | m\_potatoesindustry |
| LowPriority< FarmEvent \* > | m\_queue |
| vector< TTypesOfVegetation > | m\_rotation |
| int | m\_rotation\_sync\_index |
| SpringBarleyCloverGrass \* | m\_sbarleyclovergrass |
| SeedGrass1 \* | m\_seedgrass1 |
| SeedGrass2 \* | m\_seedgrass2 |
| SetAside \* | m\_setaside |
| SpringBarley \* | m\_springbarley |
| SpringBarleyCloverGrassStrigling \* | m\_springbarleyclovergrassstrigling |
| SpringBarleyPeaCloverGrassStrigling \* | m\_springbarleypeaclovergrassstrigling |
| SpringBarleyPTreatment \* | m\_springbarleyptreatment |
| SpringBarleySeed \* | m\_springbarleyseed |
| SpringBarleySilage \* | m\_springbarleysilage |
| SpringBarleySKManagement \* | m\_springbarleyskmanagement |
| SpringBarleyStrigling \* | m\_springbarleystrigling |
| SpringBarleyStriglingCulm \* | m\_springbarleystriglingculm |
| SpringBarleyStriglingSingle \* | m\_springbarleystriglingsingle |
| SpringRape \* | m\_springrape |
| bool | m\_stockfarmer |
| Triticale \* | m\_triticale |
| WinterBarley \* | m\_winterbarley |
| WinterBarleyStrigling \* | m\_winterbarleystrigling |
| WinterRape \* | m\_winterrape |
| WinterRapeStrigling \* | m\_winterrapestrigling |
| WinterRye \* | m\_winterrye |
| WinterRyeStrigling \* | m\_winterryestrigling |
| WinterWheat \* | m\_winterwheat |
| WinterWheatStrigling \* | m\_winterwheatstrigling |
| WinterWheatStriglingCulm \* | m\_winterwheatstriglingculm |
| WinterWheatStriglingSingle \* | m\_winterwheatstriglingsingle |
| WWheatPControl \* | m\_wwheatpcontrol |
| WWheatPToxicControl \* | m\_wwheatptoxiccontrol |
| WWheatPTreatment \* | m\_wwheatptreatment |
| YoungForestCrop \* | m\_youngforest |

---

## Detailed Description

A farm that can have its rotation defined by the user at runtime.

---

## Constructor & Destructor Documentation

|  |  |  |  |  |  |
| --- | --- | --- | --- | --- | --- |
| UserDefinedFarm3::UserDefinedFarm3 | ( | void |  | ) |  |

References Farm::m\_farmtype, Farm::m\_stockfarmer, Farm::ReadRotation(), and tof\_UserDefinedFarm3.

: Farm() // 17

{

m\_farmtype = tof\_UserDefinedFarm3;

m\_stockfarmer = true;

// This farm type reads its rotation from a special file PesticideTrialTreatment.rot

ReadRotation("UserDefinedFarm3.rot");

}

---

The documentation for this class was generated from the following files:

- farm.h
- farm.cpp


- UserDefinedFarm3
- Generated on Thu Jan 10 2013 13:15:38 for ALMaSS Skylark ODdox by
   1.8.1.1
